# Defining Pollen Seasons: Background and Recommendations

**DOI:** 10.1007/s11882-018-0829-z

**Published:** 2018-10-29

**Authors:** Katharina Bastl, Maximilian Kmenta, Uwe E. Berger

**Affiliations:** Research group Aerobiology and pollen information, Department of Oto-Rhino-Laryngology, Medical University of Vienna, Währinger Gürtel 18-20, A-1090 Wien, Austria

**Keywords:** Pollen season definition, Main pollen season, Pollen concentration, Allergen exposure, Clinical trials, Aerobiology

## Abstract

**Purpose of Review:**

The definition of a pollen season determines the start and the end of the time period with a certain amount of pollen in the ambient air. Different pollen season definitions were used for a long time including the use of different terms for data and methods used to define a pollen season. Recently suggested pollen season definitions for clinical trials were tested and applied for the first time to more aeroallergens.

**Recent Findings:**

This is a review on pollen season definitions and the latest recommendations. Recently, proposed terminology in aerobiology is promoted here in order to support reproducibility and repeatability in research. Two pollen season definitions, one based on percentages and one based on pollen concentrations, were tested.

**Summary:**

Percentage definitions can be recommended for standard aerobiological routines and for retrospective applications, whereas pollen concentrations definitions can be recommended for prospective applications such as clinical trials.

## Introduction

Defining a pollen season is a prerequisite and the first task for many research questions in aerobiology and allergy research. In addition, a well-defined pollen season is crucial for the realisation of clinical trials and aerobiological applications. Various pollen season definitions have been applied in the past. The first review with pollen season definitions as the main subject and their influence on the results was done by Jato et al. [1] showing also that the duration of a pollen season based on the same definition varies from year to year. Different expressions representing the term “pollen season” were used at that time: main pollen season, main pollen period, period of maximum pollen production, pollination period, pollination season, main pollination season, principal pollination period, effective pollen season and atmospheric pollen season ([1] and references therein). In general, there are two different methods to define a pollen season: (1) defining a percentage of the annual or seasonal pollen index as start and end day or (2) defining a certain threshold (a certain daily pollen concentration with or without a certain sum over a defined period) as start and end day. It was found that the earliest and latest start and end date varied for location and pollen type as well as between the years [1] when focusing on pollen concentration data.

Recently, different pollen season definitions based on percentages (two different definitions) and on certain thresholds (eight different definitions) were analysed also with focus on symptom data [2••] for the birch and the grass pollen season. The finding of Jato et al. [1] could be repeated: all pollen season definitions performed differently for different years and pollen types. In addition, it was shown that seasons with a higher total annual pollen index result in more spatial differences than those with a lower total annual pollen index and that symptom data is a useful data source to test pollen season definitions.

This approach was continued in a study from Karatzas et al. [3, 4•]. The recently established pollen season definitions for major aeroallergens (birch, grasses, cypress, olive, ragweed) in Europe by Pfaar et al. [5••] were tested with symptom data. The grass pollen season definition was applied to 40 pollen monitoring sites in Germany for five consecutive years and showed that the start and end criteria were stable even with missing data [3]. The birch and grass pollen season definition was then tested for Germany in three consecutive years with symptom data (nasal symptoms and medication use data) in order to test the pollen season start, end and the peak pollen period [4•]: results showed that the definitions were robust concerning the pollen concentration data and that the defined period correlated with the onset of symptom data.

It should be noted that exposure to pollen (concentrations) may vary from exposure to allergen ([6] and references therein). It is known that there is a strong regional dependency on the occurrence of allergen concentrations and that the pattern of allergen concentrations can deviate from the pattern of both pollen concentrations and symptom data though significant positive correlations were found [6]. We refer herein always to exposure to pollen (concentrations) and not allergen (concentrations).

This review presents in the following in the underlying fundament of pollen season definitions including the methods used in aerobiology to gain pollen concentration data, the terminology used, a first test application of two selected pollen season definitions to other aeroallergens and resulting recommendations.

## The Importance of Terminology

Different terminology can be misleading when it comes to definitions, such as the definition of a pollen season. A range of terms has been applied for different pollen season definitions ([1]; see above). Often terms are misinterpreted, mixed up and are not correctly used. The most frequent example concerns the expressions “pollen count” and “pollen concentration”. Pollen concentration is understood herein as daily mean pollen concentration following common practice (see Underlying methods in Aerobiology) to use its short version “pollen concentration”. Pollen count was often used although the pollen concentration was meant instead. There is an important difference between the two terms since the pollen count refers to raw data in aerobiology, while the pollen concentration is a converted value of pollen grains/m^3^ air and can be compared between analysts, pollen monitoring sites and time periods [7•]. The most important terms underlying this review are shortly described in Table 1 following Galán et al. [7•]. Those terms should be standard for the scientific community in aerobiology and are clearly defined, even though they are not exhaustive.

**Table 1.** Recently defined and recommend terminology for aerobiology and especially for defining pollen seasons as baseline herein following Galán et al. [7•]

Term (abbreviation)	Description
Annual pollen integral (APIn) Pollen *day/m^3^	Integral over time; replaces the index API and is obtained by summing the average daily concentration over the whole year or by multiplying the average concentration of the whole year
Seasonal pollen integral (SPIn) Pollen *day/m^3^	Integral over time; replaces the index SPI and is obtained by summing the average daily concentration over a given period of time or by multiplying the average concentration of the whole season by the season duration
Main pollen season (MPS)	Time period of significant pollen concentrations in the atmosphere; method of definition should be clearly stated
Pollen concentration expressed, e.g. as pollen grains/m^3^	Number of pollen grains per unit volume of air
Pollen count	Raw data of the aerobiological analysis; integer quantity that cannot be compared and needs conversion to concentrations

The annual pollen index (API) and the seasonal pollen index (SPI) were often used to compare pollen seasons. They are now replaced by the Annual Pollen Integral (APIn) and the Seasonal Pollen Integral (SPIn) respectively. The change concerns only the terminology with being more precise and leaves its calculation unaffected. The annual pollen integral takes pollen the year round into account, while the seasonal pollen integral takes pollen within a given period of time—a defined pollen season—into account (Table 1). In addition, the term main pollen season (MPS) was suggested by Galán et al. [7•] to refer to a time period of significant pollen concentrations, that needs to be clearly defined (Table 1).

As such, the definition of a pollen season is still open from a scientific point of view in aerobiology. This openness is advantageous for research in aerobiology, because pollination can vary dramatically in different countries and regions. Therefore, various definitions can be applied adapting to a regional situation and covering the needs of different work tasks. Those can be comprised of the preparation of pollen calendars, pollen forecasts and establishing pollen load levels. Pollen information services and aerobiologists can thus make use of different pollen season definitions for different tasks (see Prerequisites and Requirements for Pollen Season Definitions). However, it is crucial to agree upon a common terminology when defining a pollen season in order to ensure repeatability and reproducibility in both basic research and clinical trials. Recent work has provided a fundamental basis for this framework [7•].

## Underlying Methods in Aerobiology

The definition of a pollen season is not only influenced by terminology but also by the methods used to sample and analyse pollen data. Several hundred different pollen and spore samplers have been constructed and used in the last decades [8]. In general, there are two main sampling techniques in use in aerobiology: gravimetric and volumetric methods [9]. The gravimetric method is a simple approach to collect airborne particles on a sticky surface (e.g. microscopic slide) placed in a container to protect the surface from rain or other influences [8]. However, calculations about daily or hourly pollen concentrations are not possible due to the deposition results of the gravimetric nature. Furthermore, accurate pollen season definitions (starting and ending with a given day) are either not possible or will deviate significantly from samplers using the volumetric method.

Today, the most common method to sample pollen is the volumetric method. In Europe, the use of suction samplers of the Hirst design [10] is the standard method since decades. Other devices used for pollen sampling are cascade impactors or whirling arm samplers [8]. The advantage of a volumetric pollen sampler is the elicitation of pollen concentration data on a daily or hourly basis. Moreover, the Hirst type pollen sampler is a low-volume sampler and the intake of air can be directly compared with the intake of the human lung. Hence, pollen concentration data cannot only be compared with meteorological data but also with symptom data from patients suffering from allergic rhinitis.

Pollen monitoring stations of the Hirst design suck in 10 l per minute if adjusted correctly. A routine control of the airflow is critical since deviations from the 10 l per minute can occur depending on the trap and flow meter [11]. An aluminium drum inside the pollen trap is rotating with 2 mm per hour while exposed to the airflow. A sticky tape is wound up around the drum and catches the particles. The drum has to be changed latest after 7 days. The sticky tape is prepared in the laboratory after a drum change and the tape is cut into daily sections, which are transferred to microscopic slides. A mounting medium (Gelvatol, Mowiol, glycerol jelly or others in combination with basic fuchsine or safranin) is used to stain and fix the daily sample on the slide. The stain is used to easily discern organic material within the sample. Organic material will be coloured in a slight pink or red colour depending on the staining intensity. There are different methods for analysing daily samples such as the horizontal transvers reading method, the vertical transvers reading method and the random fields method [12]. Aerobiological samples are analysed routinely by the means of a light microscope at magnifications between × 25 and × 60, but mostly at × 40. Analysts determine and count each pollen grain in a given area of the slide (depending on the counting method used). The raw data, the pollen count, is then corrected with the conversion factor. The conversion factor includes the field of view and the percentage of the slide surface (number of horizontal/vertical transects and random fields) as well as the total amount of air sucked in.

In addition to this traditional method, there is a range of automated pollen counting devices available—most of them still in test phase and not implemented in aerobiological routines (e.g. [13–15]). There is great variability in the methods used of automated systems ranging from colour and shape information, fluorescence microscopy, flow cytometry and laser ablation mass spectrometry to Raman microscopy (see [16] for a recent summary). Therefore, precision rates vary and the number of pollen types counted varies as well depending on the method. Standardisation and scientific recommendations for handling such data has still to be awaited since there are possible pitfalls that have to be clarified first [16]: (1) precision rates have to reach a certain minimum level, (2) a wide range of pollen types has to be included in the automatization, (3) comparability to pollen data assessed by Hirst-type pollen traps (as described above) has to be assured, (4) comparison datasets (if used) have to be continuously extended and proved to allow the detection of new pollen types in a region and (5) flexibility to react on changes in the pollen spectrum and special conditions (clumped or fragmented pollen grains) has to be implemented to correctly.

Up to now, there is no standard or EU regulation containing pollen sampling and pollen analysis. However, the European Aerobiology Society (EAS) in cooperation with the European pollen database (EAN) published minimum recommendations how pollen sampling and pollen analysis should be standardised when using a sampler of the Hirst design [12]. EAN is a voluntary cooperation of local pollen monitoring networks, charities, universities, private persons and governmental entities and consists of more than 400 active pollen-monitoring stations in Europe. The quality and completeness of pollen concentration data within EAN is checked on a regular basis and makes significant contribution in European projects and the development of forecast models such as SILAM [17, 18] and CAMS [19]. Meanwhile, a technical sheet about the sampling and analysis of airborne pollen grains by use of the volumetric Hirst method was published by EAS in cooperation with EAN with the goal to become an EU standard [20]. It should be noted that all pollen season definitions herein and in the literature cited are based on daily pollen concentrations gained by Hirst-type pollen monitoring stations.

## Test of Two Different Pollen Season Definitions to Various Aeroallergens

We selected two different pollen season definitions to give an example of their applicability and functionality. The first is the standard definition included in the EAN pollen database and starts at the day with 1% of the APIn and ends at the day of 95% of the APIn. This definition was tested recently by Bastl et al. [2••] against other definitions and was developed for standard applications in aerobiology (research and routine work), herein referred to as “EAN definition”. The second definition was established by Pfaar et al. [5••], herein referred to as “EAACI definition”, and works with pollen concentrations instead of percentages and is different for different aeroallergens: (1) The birch pollen season starts on the first day of 5 days (out of seven consecutive days) with each of these 5 days ≥ 10 pollen/m^3^ and with a sum of these 5 days of ≥ 100 pollen/m^3^ and ends on the last days fulfilling the same requirements and (2) the grass and ragweed pollen season starts on the first day of 5 days (out of seven consecutive days) with each of these 5 days ≥ 3 pollen/m^3^ and with a sum of these 5 days of ≥ 30 pollen/m^3^ and ends on the last days fulfilling the same requirements. These definitions were tested by Karatzas et al. [3, 4•] in addition and were developed for clinical trials. The birch pollen season definition was applied herein to birch, hazel and alder since all of them are shrubs/trees. The grass/ragweed pollen season definition was applied to grass, mugwort and ragweed for herbaceous plants herein. It should be noted, that those definitions were developed for birch, grasses, cypress, olive and ragweed [5••] and were hitherto not applied to other aeroallergens serving here as a first test phase for more aeroallergens of importance such as hazel, alder and mugwort.

**Fig. 1:**
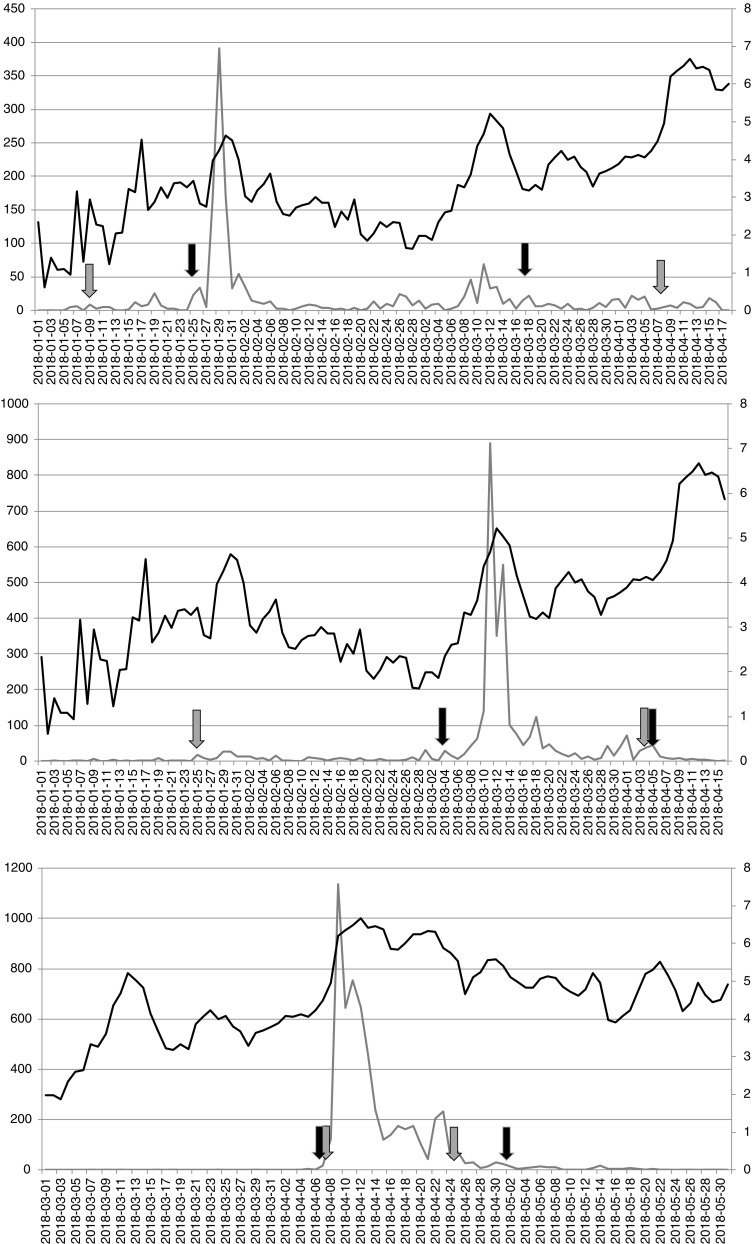
Pollen concentrations (grey curve and left y-axis; upper hazel (*Corylus*), middle alder (*Alnus*), lower birch (*Betula*)) and symptom load index (black curve and right y-axis) for important shrub/tree taxa in Austria. Data originates from Vienna for pollen concentrations and from the Pannonian Lowlands (Eastern part of Austria) for the symptom data. Grey arrows mark the EAN definition based on percentages. Black arrows mark the EAACI definition based on pollen concentrations on daily basis and a certain sum for the start and end. Note the longer duration of the pollen season based on the EAN definition except for birch

**Fig. 2:**
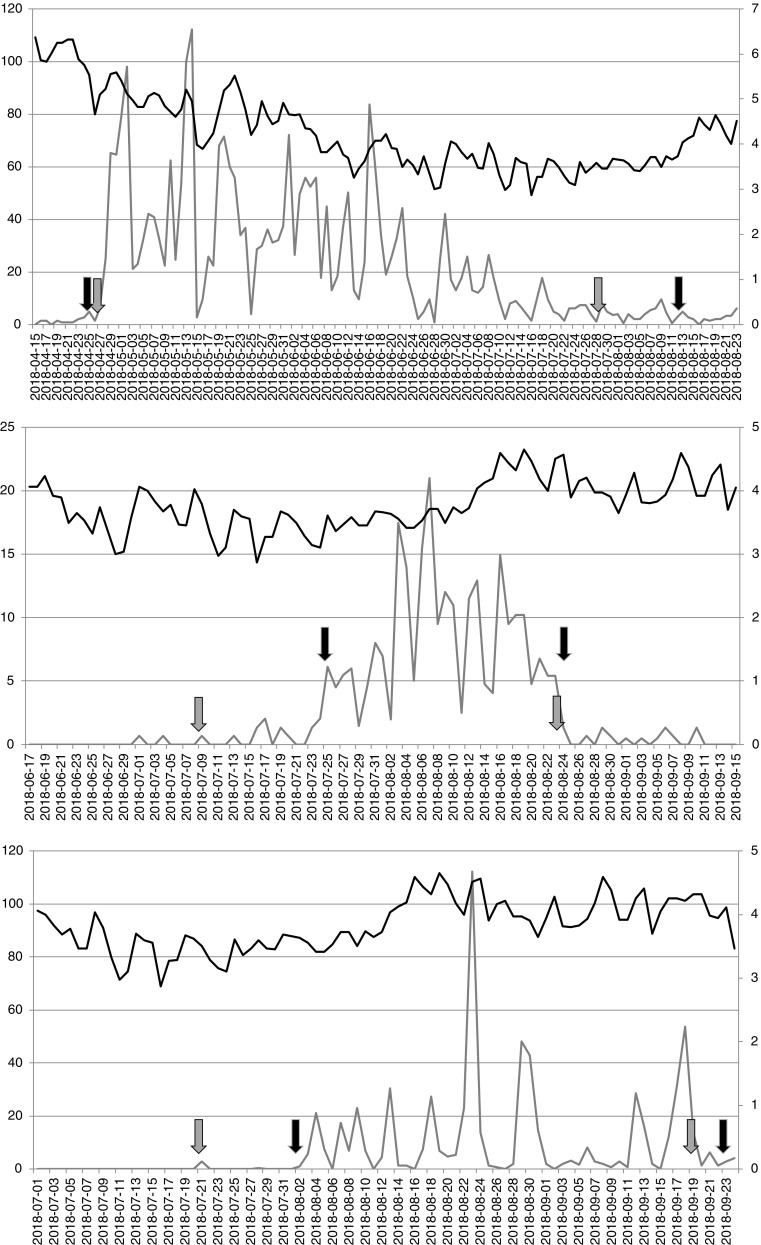
Pollen concentrations (grey curve and left y-axis; upper grasses (Poaceae), middle mugwort (*Artemisia*), lower ragweed (*Ambrosia*)) and symptom load index (black curve and right y-axis) for important herbaceous taxa in Austria. Data originates from Vienna for pollen concentrations and from the Pannonian Lowlands (Eastern part of Austria) for the symptom data. Grey arrows mark the EAN definition based on percentages. Black arrows mark the EAACI definition based on pollen concentrations on daily basis and a certain sum for the start and end. Note the longer duration of the pollen season based on the EAN definition except for grasses

Data used herein is comprised of pollen concentration data of Vienna during the year 2018. Pollen data was retrieved from the EAN database. Pollen season definitions were calculated for each of the six aeroallergens following the respective definitions (EAN definition = grey arrows; EAACI definition = black arrows; Figs. 1 and 2). Symptom data was retrieved from the patient’s hay-fever diary (as in [3, 4•] and [5••]), but was calculated as symptom load index (SLI; see [21]). It should be noted that the symptom data includes data on eye, nose and lung symptoms as well as the medication and that users fill in voluntarily and are thus not diagnosed patients. The SLI was calculated as daily mean for the whole eastern regions of Austria, the Pannonian Lowlands, to ensure high user numbers and thus a well representability due to high user numbers [22]. This short analysis shall serve as a first trial for different aeroallergens and is therefore limited to a region and the year of 2018. Results are described and discussed in the following separate for each of the selected pollen seasons.

The hazel pollen season starts on the 9th of January 2018 and ends on the 8th of April 2018 based on the EAN definition, whereas it starts on the 25th of January 2018 and ends on the 18th of March 2018 based on the EAACI definition. The hazel pollen season is longer when the EAN definition is used. The first hazel pollen concentrations are thus caught and also the first symptom in the beginning and in mid of January. The EAN definition ends after relevant pollen concentrations cease to occur. The EAACI definition catches the beginning of MPS, starts well before the peak and ends after a second small peak around mid of March (Fig. 1 upper graph). However, it should be noted that the second small peak in March coincides with the peak of the alder pollen season (see below and Fig. 1 middle graph). This late occurrence of hazel pollen can be attributed to the flower of ornamental plants, the corkscrew hazel, which is often planted in parks and in private gardens. The small and late peak is most possible relevant for hazel pollen allergy sufferers, but the question if alder pollen is more relevant at that time has to be asked. The individual situation and the possible cross-reactivity due to the close relationship of alder and hazel in the birch family should be considered as well. The application of the EAN and the EAACI definition works in this example for the hazel pollen season.

The alder pollen season starts on the 25th of January 2018 and ends on the 4th of April 2018 based on the EAN definition, whereas it starts on the 4th of March 2018 and ends on the 5th of April 2018 based on the EAACI definition. The alder pollen season is longer when the EAN definition is used. The first alder pollen concentrations are caught in the end of January/beginning of February. The EAN definition ends with the period of the last relevant occurrence of alder pollen nearly on the same day as the EAACI definition. The EAACI definition catches the MPS (Fig. 1 middle graph). The alder pollen season ends in both cases before the birch pollen season (see below), but overlaps with the hazel pollen season. This is the usual case in Austria. Each of those seasons shows a significant increase in the symptom load index during the peak of the season. The alder pollen season occurred later than on average in 2018 resulting in a break before the birch pollen season. This period is shorter than usually for the selected year 2018. The application of the EAN and the EAACI definition works in this example for the alder pollen season.

The birch pollen season starts on the 8th of April 2018 and ends on the 25th of April 2018 based on the EAN definition, whereas it starts on the 7th of April and ends on the based on the 2nd of May 2018 EAACI definition. The birch pollen season is shorter when the EAN definition is used in contrast to the case of hazel and alder. The birch pollen season started rapidly in 2018, which is usually the case in Austria. Thus, the MPS is caught by the EAN and the EAACI definition. The EAACI definition catches also the fading of the birch pollen season when the grass pollen season has already started (see below). The birch and the grass pollen season may show more or less overlaps depending on the year. However, this possible overlap should be considered especially for clinical trials and for patients who are poly-sensitised and suffer from a birch and a grass pollen allergy. The peaks in the symptom load index during end of April are most possible attributed more to the beginning of the grass pollen season than to the end of the birch pollen season. The application of the EAN and the EAACI definition works for the birch pollen season as already shown in previous publications [3, 4•] and [5••].

The grass pollen season starts on the 27th of April 2018 and ends on the 29th of July 2018 based on the EAN definition, whereas it starts on the 25th of April and ends on the 13th of August based on the EAACI definition. The grass pollen season is shorter when the EAN definition is used in contrast to the weeds mugwort and ragweed (see below). First symptoms and relevant grass pollen concentrations as well as the MPS are caught by both pollen season definitions. There is a long time period of fading grass pollen concentrations from around mid of July until the end of August due to the flower of different grass species (Fig. 2 upper graph). Different grass species may be of different importance to individual grass pollen allergy sufferers (e.g. [23]). Therefore, multiple peaks in the symptom data may vary in comparison to the grass pollen concentrations reflecting the impact of the flower of certain grass species. As an example, the peak around the 20th of May in the symptom load index is higher than around the 13th of May although the corresponding grass pollen concentrations are lower at the same time. The late end of the grass pollen season, especially for the EAACI definition, results in an overlap with the mugwort pollen season (see below) and the same possible issue as with the overlap of birch and the grass pollen season, although the frequency of mugwort pollen sensitizations in the population of Eastern Austria is much lower than for grass and birch pollen [24]. The application of the EAN and the EAACI definition works for the grass pollen season as already shown in previous publications [3, 4•] and [5••] with the only limitation that an earlier end of the season could be more beneficial in the case of the year 2018.

The mugwort pollen season starts on the 9th of July 2018 and ends on the 24th of August 2018 based on the EAN definition, whereas it starts on the 25th July 2018 and ends on the 25th August 2018 based on the EAACI definition. The mugwort pollen season is longer when the EAN definition is used. The first very low mugwort pollen concentrations are caught by the EAN definition, while the EAACI definition catches the MPS accurately. The end of the mugwort pollen season is equally caught by both definitions nearly on the same day (Fig. 2 middle graph). The symptom load index stays almost on the same level during the defined period. The symptom load index increases after the peak during the last part of the mugwort pollen season. This increase is most probable attributed to the flower of ragweed (see below), which peaked in this time. The mugwort pollen season and its progress are not well reflected in the symptom data. Explanations for this phenomenon could be a lower user number, the relative low importance of the flower of mugwort to pollen allergy sufferers in comparison with other aeroallergens, the occurrence of fungal spores in the air during summer and autumn or the overall relatively low mugwort pollen concentrations (in comparison with other pollen types). The application of the EAN and the EAACI definition works in this example for the mugwort pollen season.

The ragweed pollen season starts on the 21st of July 2018 and ends on the 19th of September 2018 based on the EAN definition, whereas it starts on the 2nd of August 2018 and ends on the 23rd of September 2018 based on the EAACI definition. The ragweed pollen season is longer when the EAN definition is used. The time period of low ragweed pollen concentrations is caught by the EAN definition. It ends after the last relevant occurrence of ragweed pollen. The EAACI definition catches the MPS, starts before the first significant ragweed pollen concentrations and ends with low ragweed pollen concentrations. The peak period is reflected in the symptom load index (Fig. 2 lower graph). The mugwort and the ragweed pollen season overlap. However, the symptom load index indicates a higher burden during the flower of ragweed. It remains unknown if the burden from ragweed pollen is higher due to higher pollen concentrations despite lower prevalence [24] or if cross-reactivity between both pollen types and the overlap of both seasons could be the explanation of this phenomenon. The application of the EAN and the EAACI definition works for the ragweed pollen season as well.

Summarising both pollen season definitions used, one based on percentages and one based on pollen concentrations, work well in this example to catch the concerned period of relevant pollen concentrations. The EAACI pollen season definition is more likely to focus on the MPS, whereas the EAN pollen season definition is more likely to focus on the whole period of low, but relevant pollen concentrations.

## Prerequisites and Requirements for Pollen Season Definitions

The tools and terminology used have to be clearly stated when a pollen season is defined. There should be no doubt about the meaning of terms and about the methods used in order to assure reproducibility and repeatability no matter for which task (aerobiological routine or clinical trials). We recommend using the recently defined terminology [7•]; see Table 1 for an excerpt of most important terms) to avoid misunderstandings.

The completeness of pollen data used influences the resulting start and end day of a pollen season. Pollen concentrations that were not measured cannot be considered. Therefore, data gaps are a factor, which has to be considered when selecting pollen data. The most complete a pollen dataset is the better for the pollen season definition. In the test described herein, ragweed pollen data was considered until the 30th of September (because more data was not available by that time). Defining the ragweed pollen season in December with data from October and November might shift the start and end day.

Furthermore, the exact pollen season definition should be selected with care due to a range of possible pitfalls among them: (1) the influence of overlapping preceding or successive pollen seasons; (2) the purpose of the research question (are low pollen concentrations relevant or is the MPS in focus?) and (3) symptom behaviour of persons concerned, if relevant (e.g. during the flower of different grass species, cross-reactivity). The pollen season definition chosen should consider all mentioned issues.

Our findings are limited to pollen data gained by Hirst-type pollen traps and routine aerobiological analyses following the minimum recommendations [12]. It has to be awaited if and how other data types gained, e.g. by automated pollen samplers can be used for such applications. The burden of the persons concerned and the responsibility of research in this field and institutions entrusted with pollen information has to be upheld in any case to avoid any possible harm to pollen allergy sufferers [16].

## Conclusions

The definition of a pollen season is relevant since it may influence the results in research or the concerned application. A pollen season definition has to be formulated clearly, in best case in an agreed terminology [7•], and be based on robust methods including high-quality pollen data, no matter if the APIn, SPIn or pollen concentrations are used.

Pollen data based on scientific standards has to be the fundament of pollen season definitions. Such high-quality data is more difficult to receive since the financing of pollen monitoring stations is becoming more and more challenging nowadays. The EAN database has proved as a highly valuable tool and was the basis for most of the recent studies on pollen season definitions [2••, 4•, 5••]. Up-to-date, there is no other possibility than to use pollen data gained by the current measuring technique (Hirst-type pollen traps and minimum recommendations), which is standardised and scientifically robust. The quality of pollen data has to be taken into account at all times especially before it is used for applications that depend on accurateness, such as defining a pollen season.

The aim of the question/hypothesis determines the possible pollen season definition selected. The EAN definition proved herein and in Bastl et al. [2••] highly useful if also low pollen concentrations and the whole period of the presence of an aeroallergen should be considered. Therefore, it can be recommended for aerobiological routine work such as pollen calendars and pollen season analysis. However, it is only applicable retrospectively when pollen data is available for the whole season.

The EAACI definition [5••] proved highly useful when the MPS is in focus and a high, continuous exposure to a specific aeroallergen has to be assured and a fast communication of the start/end of a pollen season is required. Therefore, this definition is recommended for clinical trials, because it works prospectively as well. Additionally, it has been shown that the birch pollen season definition works for hazel and alder and that the grass/ragweed pollen season definition works for mugwort as well. The example is limited to a certain region and to 1 year, so it should be analysed in more detail in future if the EAACI pollen season definitions should be applied to other aeroallergens as well (e.g. ash (*Fraxinus*)). Interestingly, the average symptom load index for a season is about on the same level for the EAN definition and the EAACI definition (definition 1 for EAN and 8 for EAACI in [2••]) proposing a sufficient comparability between both for certain assessments.

Finally, the selection of a pollen season definition depends on the specific needs of the research question and deviations from already used and recommended definitions may be justified if certain requirements exist (e.g. regional framework, study design) and as long as methods and terminology used are always clear and precise.

## Abbreviations

EAN: European Aeroallergen Network
MPS: main pollen season
PHD: patient’s hay-fever diary
SPIn: seasonal pollen integral.

## References

[1] Jato V, Rodriguez-Rajo FJ, Alcázar P, De Nuntiies P, Galán C, Mandrioli P (2006). May the definition of pollen season influence aerobiological results?. Aerobiologia.

[2] Bastl K, Kmenta M, Geller-Bernstein C, Berger U, Jäger S (2015). Can we improve pollen season definitions by using the symptom load index in addition to pollen counts?. Environ Pollut.

[3] Karatzas K, Riga M, Berger U, Werchan M, Pfaar O, Bergmann KC (2018). Computational validation of the recently proposed pollen season definition criteria. Allergy.

[4] Karatzas K., Katsifarakis N., Riga M., Werchan B., Werchan M., Berger U., Pfaar O., Bergmann K.-C. (2018). New European Academy of Allergy and Clinical Immunology definition on pollen season mirrors symptom load for grass and birch pollen-induced allergic rhinitis. Allergy.

[5] Pfaar O, Bastl K, Berger U, Buters J, Calderon MA, Clot B (2017). Defining pollen exposure times for clinical trials of allergen immunotherapy for pollen-induced rhinoconjunctivitis – an EAACI position paper. Allergy.

[6] Bastl K, Kmenta M, Pessi AM, Prank M, Saarto A, Sofiev M (2016). First comparison of symptom data with allergen content (Bet v 1 and Phl p 5 measurements) and pollen data from four European regions during 2009–2011. Sci Total Environ.

[7] Galán C, Ariatii A, Bonini M, Clot B, Crouzy B, Dahl A (2017). Recommended terminology for aerobiological studies. Aerobiologia.

[8] Rantio-Lehtimäki A. Sampling Airborne Pollen and Pollen Antigens. In: D’Amato G, Spieksma FTM, Bonini S, editors. Allergenic Pollen and Pollinosis in Europe. Blackwell Scientific Publications, 18–23; 1991.

[9] Raynor GS. Sampling techniques. In: Edmonds RL, editor. Aerobiology. The ecological system approach. US/IBP Synthesis Series. 10:151–169; 1979.

[10] Hirst JM (1952). An automatic volumetric spore trap. Ann Appl Biol.

[11] Oteros J, Buters J, Laven G, Röseler S, Wachter R, Schmidt-Weber C, Hofmann F (2017). Errors in determining the flow rate of Hirst-type pollen traps. Aerobiologia.

[12] Galán C, Smith M, Thibaudon M, Frenguelli G, Oteros J, Gehrig R (2014). Pollen monitoring: minimum requirements and reproducibility of analysis. Aerobiologia.

[13] Crouzy B, Stella M, Konzelmann T, Calpini B, Clot B (2016). All-optical automatic pollen identification: towards an operational system. Atmos Environ.

[14] Ronneberger O, Schultz E, Burkhardt H (2002). Automated pollen recognition using 3D volume images from fluorescence microscopy. Aerobiologia.

[15] Wetzlar H. Pollen Monitor BAA500. Datasheet 2016. 2016; Accessed 2nd October 2018. http://www.hund.de/images/pdf/Datasheet_BAA_english_12.03.09.pdf.

[16] Bastl K, Berger M, Bergmann KC, Kmenta M, Berger U (2017). The medical and scientific responsibility of pollen information services. Wien Klin Wochenschr.

[17] Siljamo P, Sofiev M, Filatova E, Grewling L, Jäger S, Khoreva E, Linkosalo T, Ortega Jimenez S, Ranta H, Rantio-Lehtimäki A, Svetlov A, Veriankaite L, Yakovleva E, Kukkonen J (2012). A numerical model of birch pollen emission and dispersion in the atmosphere. Model evaluation and sensitivity analysis. Int J Biometeorol.

[18] Sofiev M, Siljamo P, Ranta H, Linkosalo T, Jäger S, Rasmussen A (2013). A numerical model of birch pollen emission and dispersion in the atmosphere. Description of the emission module. Int J Biometeorol.

[19] Sofiev M, Berger U, Prank M, Vira J, Arteta J, Belmonte J, Bergmann KC, Chéroux F, Elbern H, Friese E, Galan C, Gehrig R, Khvorostyanov D, Kranenburg R, Kumar U, Marécal V, Meleux F, Menut L, Pessi AM, Robertson L, Ritenberga O, Rodinkova V, Saarto A, Segers A, Severova E, Sauliene I, Siljamo P, Steensen BM, Teinemaa E, Thibaudon M, Peuch VH (2015). Macc regional multi-model ensemble simulations of birch pollen dispersion in Europe. Atmos Chem Phys.

[20] Thibaudon M, Monnier S. Ambient Air – Sampling and analysis of airborne pollen grains and fungal spores for networks related to allergy – volumetric Hirst method. Programme and abstract book of the 11th International Congress on Aerobiology, Parma, Italy, p.112. 3rd – 7th September 2018.

[21] Bastl K, Kmenta M, Jäger S, Bergmann KC, Berger U, EAN (2014). Development of a symptom load index: enabling temporal and regional pollen season comparisons and pointing out the need for personalized pollen information. Aerobiologia.

[22] Bastl K, Berger U, Kmenta M (2016). Ten questions about pollen and symptom load and the need for indoor measurements in built environment. Build Environ.

[23] Kmenta M, Bastl K, Kramer MF, Hewings SJ, Mwange J, Zetter R, Berger U (2016). The grass pollen season 2014 in Vienna: a pilot study combining phenology, aerobiology and symptom data. Sci Total Environ.

[24] Hemmer W, Schauer U, Trinca AM, Neumann C. Endbericht 2009 zur Studie: Prävalenz der Ragweedpollen-Allergie in Ostösterreich. Amt der NÖ Landesregierung. Landesamtsdirektion, Abteilung Gebäudeverwaltung, Amtsdruckerei, St. Pölten; 2010.

